# Cy3-ATP labeling of unfixed, permeabilized mouse hair cells

**DOI:** 10.1038/s41598-021-03365-x

**Published:** 2021-12-13

**Authors:** Itallia V. Pacentine, Peter G. Barr-Gillespie

**Affiliations:** Oregon Hearing Research Center & Vollum Institute, Mail Code L335A, Oregon Health & Science University, 3181 SW Sam Jackson Park Road, Portland, OR 97239 USA

**Keywords:** Cochlea, Hair cell, Inner ear, Cytoskeletal proteins, Actin, Motor proteins, Myosin

## Abstract

ATP-utilizing enzymes play key roles in hair bundles, the mechanically sensitive organelles of sensory hair cells in the inner ear. We used a fluorescent ATP analog, EDA-ATP-Cy3 (Cy3-ATP), to label ATP-binding proteins in two different preparations of unfixed hair-cell stereocilia of the mouse. In the first preparation, we lightly permeabilized dissected cochleas, then labeled them with Cy3-ATP. Hair cells and their stereocilia remained intact, and stereocilia tips in rows 1 and 2 were labeled particularly strongly with Cy3-ATP. In many cases, vanadate (V_i_) traps nucleotides at the active site of myosin isoforms and presents nucleotide dissociation. Co-application with V_i_ enhanced the tip labeling, which is consistent with myosin isoforms being responsible. By contrast, the actin polymerization inhibitors latrunculin A and cytochalasin D had no effect, suggesting that actin turnover at stereocilia tips was not involved. Cy3-ATP labeling was substantially reduced—but did not disappear altogether—in mutant cochleas lacking MYO15A; by contrast, labeling remained robust in cochleas lacking MYO7A. In the second preparation, used to quantify Cy3-ATP labeling, we labeled vestibular stereocilia that had been adsorbed to glass, which demonstrated that tip labeling was higher in longer stereocilia. We found that tip signal was reduced by ~ 50% in *Myo15a*^*sh2/sh2*^ stereocilia as compared to *Myo15a*^*sh2*^/+stereocilia. These results suggest that MYO15A accounts for a substantial fraction of the Cy3-ATP tip labeling in vestibular hair cells, and so this novel preparation could be utilized to examine the control of MYO15A ATPase activity in situ.

## Introduction

Myosin motor proteins are essential to the development of the inner ear’s actin-based stereocilia, which project from the apical surface of hair cells and detect mechanical stimuli^1^. Stereocilia are constructed with bundled actin filaments, and are enveloped by the plasma membrane. The ~ 100 stereocilia making up a hair bundle, the mechanosensory organelle of inner-ear hair cells, are coupled together with a variety of extracellular linkages. The most famous of these links is the tip link, which gates the mechanotransduction channel that underlies hair-cell transduction^2,3^. Maintaining proper tension in tip links is essential for gating the mechanotransduction channel, which underlies auditory and vestibular sensation^4^.

Myosin isoforms that move to the plus ends of actin filaments and are expressed in hair cells include MYO1C, MYO3A, MYO3B, MYO7A, and MYO15A; these isoforms transport essential structural and functional proteins from the soma into the specialized environment of the stereocilia^5^. In addition, the minus-end-directed myosin motor MYO6 also plays developmental and functional roles in stereocilia^5^. MYO7A has been proposed to interact directly or indirectly with PCDH15 and CDH23, the two cadherins that make up the hair cell’s tip links, and transports them along the stereocilia actin core to tension these linkages^6,7^. Myosins also provide a slow adaptation mechanism that reduces mechanotransduction in response to a sustained activity^8–10^, although the molecular identity of the responsible myosin remains controversial^10–14^.

Considering their essential role in hearing and balance, we sought to study the dynamic activity of myosin motor proteins in stereocilia. Most studies of myosin activity are conducted in vitro using single actin filaments as platforms for myosin proteins; these experiments do not allow localization within a cell. Antibody labeling is the usual approach for studying the localization of proteins, but typically requires fixation of the tissue; fixation causes protein crosslinking, which then prevents study of dynamic protein trafficking or movement. Direct measurement of myosin ATPase activity in isolated stereocilia offers another approach, but does not allow localization of responsible myosin isoforms^15^.

**Figure 1:**
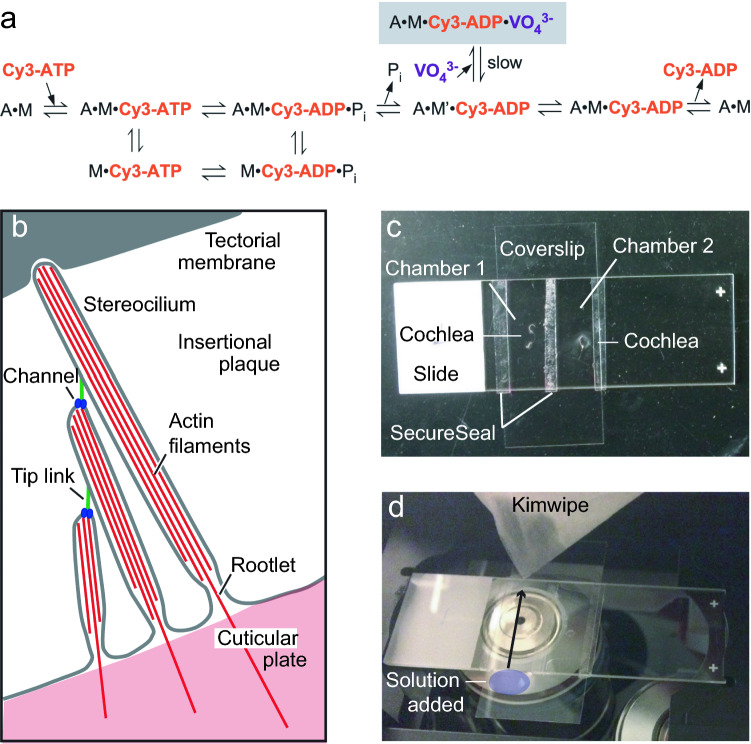
Experimental materials and apparatus. (**a**) We expect that the hydrolysis of Cy3-ATP proceeds according to this scheme, which is thought to hold for most myosin isoforms. In this scheme, actin is represented by A and myosin by M. Nucleoside triphosphates such as Cy3-ATP bind to (1) and dissociate (2) the actomyosin complex. After hydrolysis of the nucleotide (4), actin and myosin reassociate (5). A fraction of the nucleotide is hydrolyzed without dissociating the actomyosin complex (3). The hydrolysis products, inorganic phosphate (P_i_) and Cy3-ADP, are released sequentially (6) and (9). Between the release of P_i_ and that of Cy3-ADP, the myosin proceeds through a power stroke and a kinetically irreversible step (8). Vanadate can bind before Cy3-ADP release; the slow dissociation of vanadate will trap Cy3-ADP on myosin for a prolonged time (7). Although specific rate constants may differ, this mechanism is thought to hold for all myosin isoforms^34^. (**b**) Diagram of a cochlear hair bundle, with key structures called out. (**c**) Perfusion chamber. (**d**) Perfusion chamber mounted on microscope, with a tissue paper wicking liquid out of the chamber on one side and drawing liquid (blue) into the chamber following the direction of the arrow.

In previous work^9,16^, we showed that myosin molecules could be directly labeled with [^32^P]UTP if vanadate trapping^17,18^ was used to stabilize the myosin-nucleotide complex prior to photoaffinity labeling. We reasoned that vanadate trapping could also be used to slow the dissociation of fluorescent nucleotides from the active sites of myosin molecules (Fig. 1a). Moreover, if maintained in situ in a permeabilized cell preparation, fluorescent nucleotide labeling would provide an alternative way of localizing myosins within stereocilia^19^. Here, we show that EDA-ATP-Cy3 (Cy3-ATP) can be used to label unfixed, permeabilized hair cells at the tips of their stereocilia, and that a substantial proportion of the labeling is dependent on the presence of MYO15A.

## Results

We first visualized Cy3-ATP in mouse cochleas. A diagram of a prototypical hair bundle is presented in Fig. 1b. We dissected juvenile mouse cochleas and permeabilized for 10 min using a mixture of saponin and Triton X-100 at low concentration in an intracellular-like solution. We then incubated tissues with phalloidin labeled with Alexa Fluor 488, which allowed us to visualize stereocilia, and mounted the cochleas in a perfusion chamber (Fig. 1c,d). We introduced 5 μM Cy3-ATP in intracellular solution to the preparation, then imaged the hair cells using light microscopy with Airyscan detection or lattice SIM acquisition and processing.

**Figure 2:**
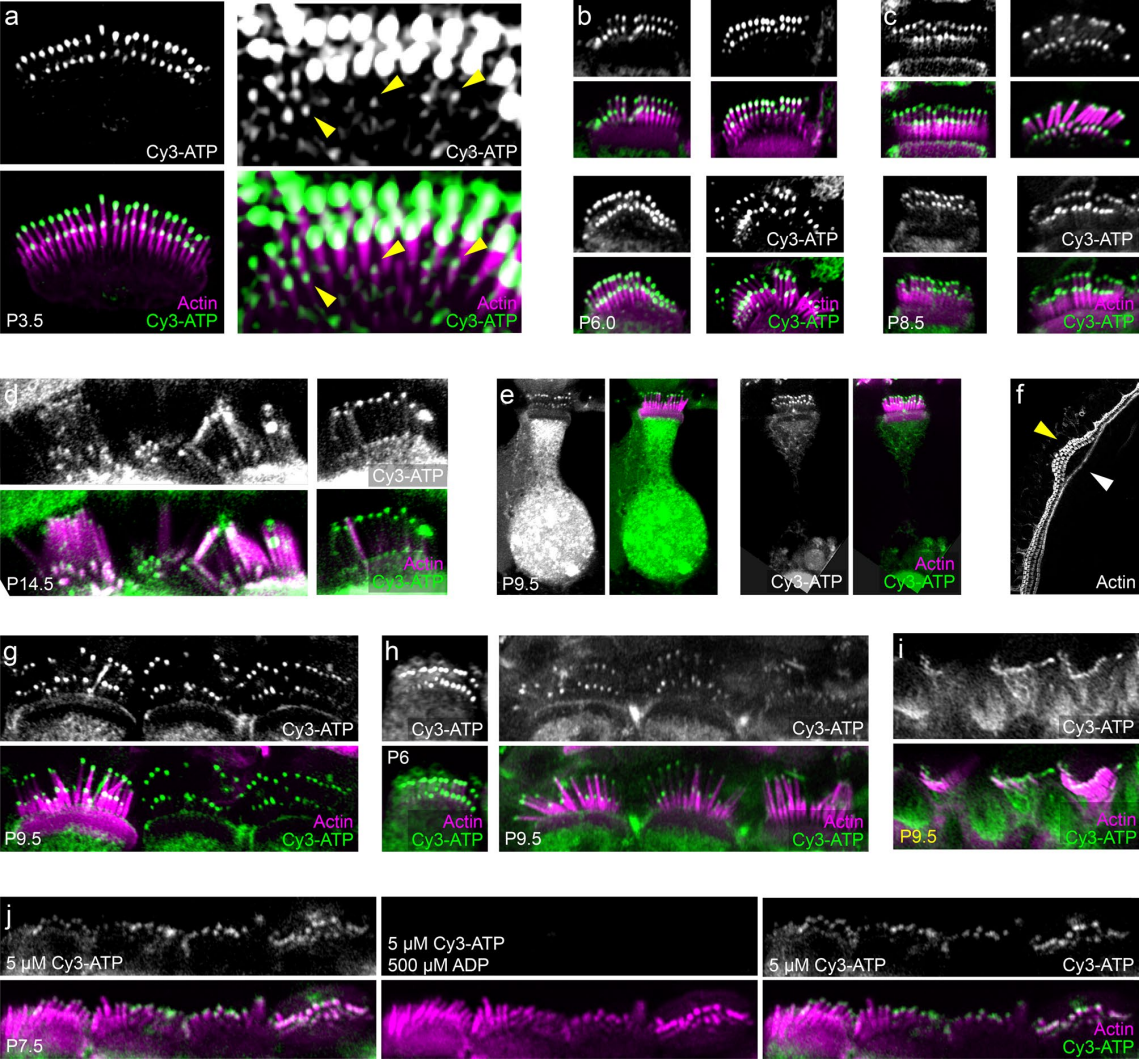
Cy3-ATP labels the tips of cochlear stereocilia. (**a**) IHCs labeled with Cy3-ATP at P3.5. Z-projections using maximum intensity. Increasing the image gain (right) shows that shorter stereocilia also have signal at their tips (yellow arrowheads). (**b**) IHCs labeled at P6.5. The top two examples are single section images; the bottom two examples are z-projections using maximum intensity. (**c**) IHCs labeled at P8.5. The top left example is a single section image; the other three examples are z-projections using maximum intensity. (**d**) IHCs at P14.5; z-projections at maximum intensity. (**e**) IHCs at P9.5; z-projections using maximum intensity. (**f**) Cochlear tissue at P9.5 labeled with phalloidin alone; z-projections using maximum intensity. The single row of inner hair cells (white arrowhead) shifted away from the three rows of outer hair cells (yellow arrowhead) in one region. (**g**, **h**) IHCs at P6 or P9.5; z-projections using maximum intensity. (**i**) OHCs labeled at P9.5; z-projections using maximum intensity. (**j**) IHCs labeled at P7.5; z-projections using maximum intensity. Left images are before exposure to ADP, middle images are during exposure to 500 μM ADP, and right-images are after ADP has been washed out. Panel full heights: (**a**) 9 μm (left) and 3.5 μm (right); (**b**,**c**) 6.7 μm; (**d**) 9.3 μm; (**e**) 40 μm; (**f**) 399 μm; (**g**–**i**) 9.3 μm; (**j**) 7.0 μm. Panel (**a**) is from a lattice-SIM processed images; all others are Airyscan. Actin was labeled with phalloidin 488. All images except f were taken during exposure to 5 μM Cy3-ATP in C57B/6 mice.

We observed consistent strong labeling of the distal tips of hair cell stereocilia (Fig. 2). While tip signal was observed in both inner (Fig. 2a–h) and outer hair cells (Fig. 2i), we focused on inner hair cells (IHCs) because their stereocilia are larger and are more easily distinguished. Moreover, IHC stereocilia often could be observed in an image plane that excluded the cell bodies, which improved our ability to measure signals at the tips. The tip signal was easily distinguished from background signal even with 5 μM Cy3-ATP in solution, suggesting that the local concentration of Cy3-ATP at tips was substantially more than 5 µM; subsequently, all cochlear images were taken during exposure to Cy3-ATP unless otherwise stated.

We examined labeling of cochlear tissue from P3.5 to P14.5 (Fig. 2a–d) and observed consistent, punctate label at the tips of stereocilia at all ages. Labeling of longer and thicker IHC stereocilia in rows 1 and 2 was considerably stronger than that in row 3 (Fig. 2a, arrowheads). By P14.5, IHCs were more resistant to permeabilization (Fig. 2d; see below). We also observed high signal from the soma, particularly the nuclei (Fig. 2e). We sometimes observed that the apical surface of the hair cell had stretched away from the cell body (Fig. 2e, left), sometimes seeming to break off entirely (Fig. 2e, right). We presume this is due to tissue perturbance by the permeabilization step.

Consistent Cy3-ATP labeling was beset by several challenges. The first was the proximity of the IHCs to the outer hair cells (OHCs); if splayed IHC stereocilia overlapped other cells, the signal from the underlying soma could overwhelm the tip signal (Fig. 2h). We found that when the cochlear tissue was perturbed sufficiently to separate the row of IHCs (Fig. 2f, white arrowhead) from the rows of OHCs (Fig. 2f, yellow arrowhead), IHCs were much more likely to be ideally oriented for imaging.

A second challenge was that unfixed IHCs were more resistant to permeabilization than other cells. Too much detergent destroyed cells; too little resulted in IHCs that were unlabeled by phalloidin but paradoxically still showed Cy3-ATP labeling (Fig. 2g). Moreover, the older the tissue, the more resistant the IHCs were to detergent. Due to these constraints, the most robust Cy3-ATP labeling of IHCs was seen between P3.5 and P8.5. Use of 0.008% saponin alone allowed selective labeling of OHCs; addition of small amounts of Triton X-100 (0.012–0.016%) to the saponin mixture was required for robust IHC labeling. Younger tissue (P3.5–P5.5) required less Triton X-100 for permeabilization (0.012%), while older tissue (P6.5–P14.5) required more (0.016%).

To confirm that our signal was a result of nucleotide binding and not due to an effect of the Cy3 tag, we co-applied an excess of ADP with Cy3-ATP (Fig. 2j). We first exposed the tissue to Cy3-ATP alone and imaged the cells, observing tip signal (Fig. 2j, left panels). We then perfused with intracellular solution to eliminate remaining Cy3-ATP; after the tip signal was below detection, we then exposed the cells to 5 µM Cy3-ATP and 500 µM ADP. We imaged the same set of cells and observed that the fluorescence signal was absent from stereocilia (Fig. 2j, middle panels). To confirm reversibility, we washed the tissue again, waited 5 min, and then re-applied 5 µM Cy3-ATP, which restored the tip signal (Fig. 2j, right panels). This experiment suggested that the binding of Cy3-ATP to stereocilia results from interaction of nucleotides with an unknown partner.

### Vanadate sensitivity of cochlear Cy3-ATP target

As assessed by protein mass spectrometry, ATP-binding molecules in vestibular hair bundles fall into several classes that exhibit widely varying concentrations^20^. Actin isoforms are very abundant, accounting for 400,000 molecules per stereocilium. The unconventional myosins MYO1C, MYO1H, MYO6, MYO7A, and MYO15A together account for about 2000 molecules per stereocilium, while the Ca^2+^ pump ATP2B2 is also present at 2000 molecules per stereocilium. A variety of other ATP-binding proteins can also be detected in stereocilia^21^.

To narrow down which proteins account for Cy3-ATP labeling at hair-cell stereocilia tips, we used orthovanadate (VO_4_^3−^, also known as vanadate), which acts as an analog of inorganic phosphate and binds to both actin and myosin^17,18,22,23^. When bound to myosins, vanadate prolongs the ADP-bound state by replacing the hydrolyzed phosphate, preventing ADP dissociation.

**Figure 3:**
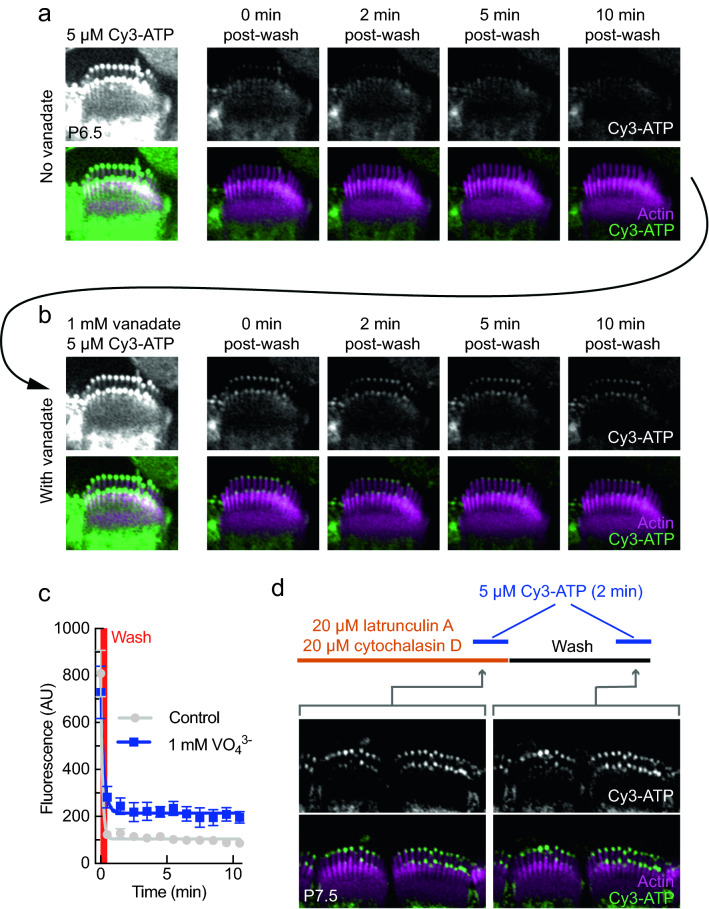
Cy3-ATP tip signal is sensitive to vanadate trapping and is not binding to actin. Actin was stained with phalloidin-488. All images are Airyscan in C57B/6 mice. (**a**) A single inner hair cell during a time-lapse experiment, z-projections with averaged intensity. (**b**) Images of the same hair cell during a second time-lapse experiment performed 5 min after the experiment in (**a**), with 1 mM vanadate in the initial solution. (**c**) Graphs of fluorescence signal at the tips of stereocilia from the cell in (**b**) and (**c**), n = 7 stereocilia at 12 time points for two conditions (with or without vanadate). The full experiment described in b and c was repeated with similar results (N = 2 mice). Statistical significance was determined by multiple t-tests with a two-stage step-up method^50^; Q = 1%. *p < 0.001 **p < 0.0001. (**d**) Single section images of two inner hair cells during an exposure and wash regiment. The orange bar represents 12 min of exposure to a co-application of 20 μM latrunculin A and 20 μM cytochalasin D. The black bar represents 8 min of wash with intracellular solution (1 min to perform wash, 5 min wait for cells to clear, 2 min with concurrent exposure to Cy3-ATP). The blue bar represents 2 min of exposure to 5 μM Cy3-ATP. N = 6 mice. Scale: All panel heights are 8.9 μm.

To test whether our observed tip signal is sensitive to vanadate, we exposed the tissue to Cy3-ATP for 2 min. We imaged during exposure, then washed and imaged the cells over 10 min (Fig. 3a). We included 250 μM ADP in our initial wash solution to prevent Cy3-ATP rebinding. We then washed with a solution lacking ADP and waited 5 min for target sites to clear. We then repeated the Cy3-ATP labeling procedure on the same preparation, this time with 1 mM vanadate co-applied with Cy3-ATP (Fig. 3b). Co-application with vanadate increased the persistent tip signal generated by Cy3-ATP (Fig. 3b-c). This result suggests that the binding partner of Cy3-ATP is sensitive to vanadate.

### No effect of actin polymerization inhibitors on cochlear Cy3-ATP tip labeling

Labeling at stereocilia tips, where actin polymerizes, suggested that newly added actin monomers might be responsible for Cy3-ATP labeling at tips. In this scenario, exchange of bound unlabeled nucleotide for Cy3-ATP might be enhanced on actin monomers found at stereocilia tips, which could add to existing filaments. To prevent actin polymerization, we used two known blockers of actin addition to pre-existing filaments, latrunculin A and cytochalasin D. Latrunculin A binds to actin monomers and prevents them from binding to actin filaments^24^, while cytochalasin D binds to the growing (positive) end of filaments, preventing monomers from binding^25^. Cochlear cells were exposed for 10 min to a mixture of 20 μM latrunculin A and 20 μM cytochalasin D, then 5 μM Cy3-ATP was added for 2 min (Fig. 3d, left panels). Cells were washed for 5 min, then re-exposed to 5 μM Cy3-ATP for 2 min. Latrunculin A and cytochalasin D had no effect on the signal at stereocilia tips (Fig. 3d). This experiment suggests that the Cy3-ATP signal is unlikely to derive from actin incorporation.

### Cy3-ATP tip labeling in isolated utricular stereocilia

To provide a better preparation for quantifying Cy3-ATP binding, we isolated vestibular stereocilia on glass^26,27^ and measured Cy3-ATP tip signal by fluorescence microscopy. Using C57BL/6 mice, utricular stereocilia mice can be isolated in much greater numbers and with much less contamination than can cochlear stereocilia. Unlike with cochlear stereocilia, when we imaged vestibular stereocilia in the presence of 5 µM Cy3-ATP in solution, the signal at tips was relatively weak compared with the solution signal. We therefore added vanadate to prolong signal lifetime, allowing robust Cy3-ATP tip signal to persist for over an hour after washing, which permitted imaging of many stereocilia with a single exposure to Cy3-ATP.

**Figure 4:**
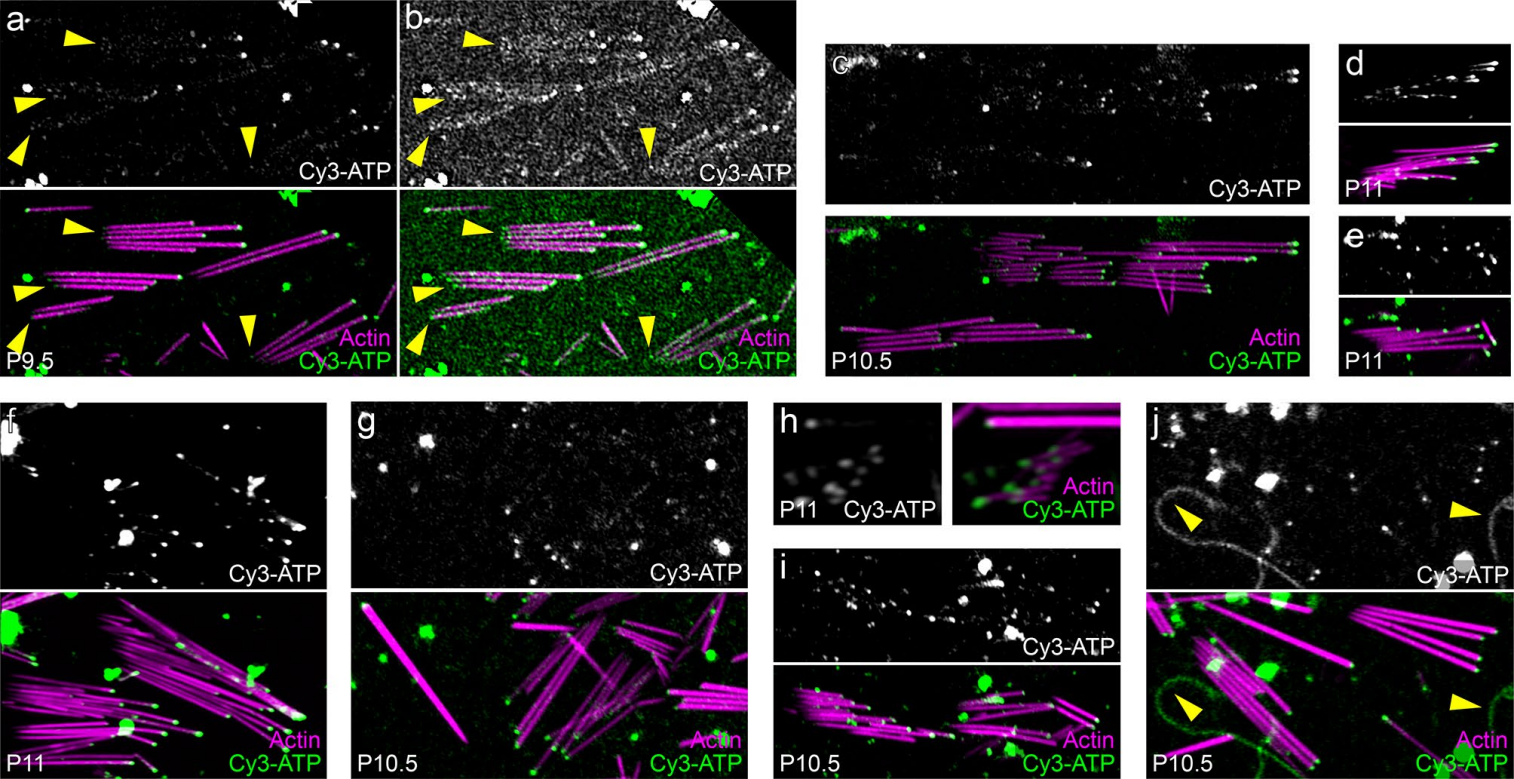
Cy3-ATP labeling in isolated utricle stereocilia. (**a**–**j**) Examples of isolated stereocilia from utricles of P9.5-P11.5 C57B/6 mice using Airyscan imaging. (**a**,**b**) show low- and high-gain images of a single field from P9.5 utricle. Yellow arrowheads indicate weak Cy3-ATP signal at stereocilia taper regions. Yellow arrowheads in (**j**) indicate probable kinocilia. Panel full heights: (**a**, **b**) and (**f**,**g**) 14 μm; c, 12 μm; (**d**,**e**) 5.8 μm; (**h**) 3.4 μm; (**i**) 8.3 μm; (**j**) 13.6 μm.

In isolated utricular stereocilia, we also observed Cy3-ATP signal at the tips of stereocilia, including even in short stereocilia (Fig. 4a–j). There was also a much less intense but consistently observable Cy3-ATP signal at the stereocilia taper region (Fig. 4a,b, yellow arrowheads), which required increasing the gain to visualize clearly (Fig. 4b). We also observed long structures that we presume to be kinocilia, as they labeled with Cy3-ATP but not phalloidin (Fig. 4j, yellow arrowheads).

### Cochlear Cy3-ATP tip labeling in myosin knockout lines

Myosin motor proteins are vanadate-sensitive, and several unconventional myosin isoforms are found in stereocilia^5^. Several are found at stereocilia tips, including MYO15A^28^. To test the role of MYO15A, we used the *shaker-2* allele of *Myo15a*^29^. Loss of MYO15A causes stunted stereocilia lengthening and reduced pruning, resulting in short hair bundles and extra rows of stereocilia.

**Figure 5:**
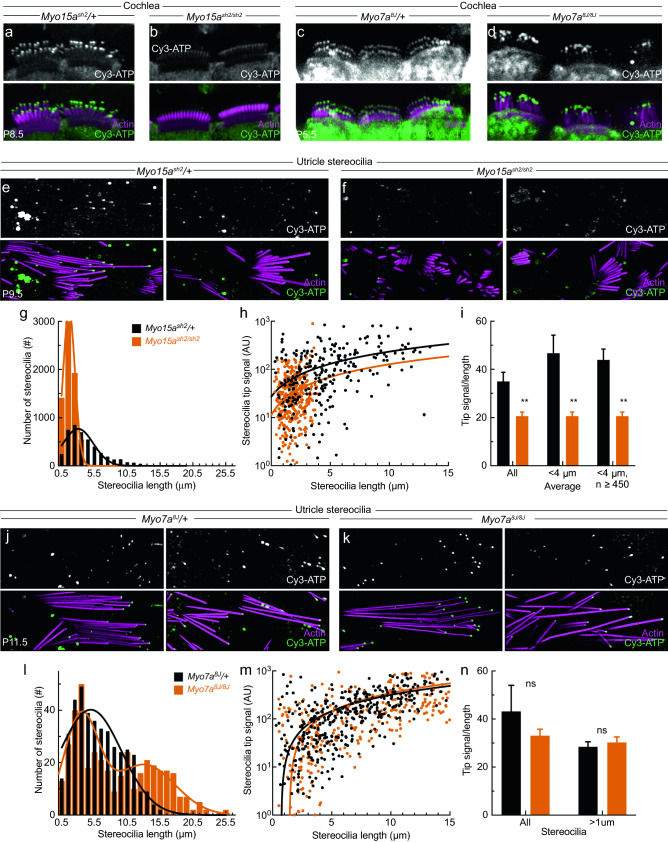
Cy3-ATP signal is significantly reduced in *Myo15a*^*sh2/sh2*^ mice, but not *Myo7a*^*8J/8J*^ mice. (**a**–**d**) Maximum intensity z-projections of cochlear hair cells. (**a**) IHCs from *Myo15a*^*sh2*^/+mice. (**b**) IHCs from *Myo15a*^*sh2/sh2*^ mice. (**c**) IHCs from *Myo7a*^*8J*^/+ mice. (**d**) IHCs from *Myo7a*^*8J/8J*^ mice. (**e**) Stereocilia isolated from *Myo15a*^*sh2*^/+ mouse utricles. (**f**) Stereocilia isolated from *Myo15a*^*sh2/sh2*^ mouse utricles. (**g**–**i**) Quantitation of utricle stereocilia from 5 *Myo15a*^*sh2*^/+ animals and 4 *Myo15a*^*sh2/sh2*^ animals. (**g**) Number of stereocilia binned by length of stereocilium (1 μm bins); single Gaussian fits. *Myo15a*^*sh2*^/+: R^2^ = 0.8694, *Myo15a*^*sh2/sh2*^: R^2^ = 0.9956. (**h**) Tip signal dependence on length for individual stereocilia. Y-axis is logarithmic; all negative values were converted to 0.1 (35 converted to 0.1 of n = 300 for *Myo15a*^*sh2*^/+; 99 converted of n = 450 for *Myo15a*^*sh2/sh2*^). Data were fit with linear regression: *Myo15a*^*sh2*^/+, 21x + 27 (R^2^ = 0.1754); *Myo15a*^*sh2/sh2*^, 12x + 11 (R^2^ = 0.0284). (**i**) Tip signal divided by length (mean ± SEM). Statistical significance determined by multiple t-tests with a two-stage step-up method^50^; Q = 1%. **p < 0.0001. Negative values were converted to zeros (*Overall*: 35 converted of n = 298 for *Myo15a*^*sh2*^/+, 99 converted of n = 450 for *Myo15a*^*sh2/sh2*^; *Overall *< *4 μm*: 25 converted of n = 143 total *Myo15a*^*sh2*^/+, 99 converted of n = 450 for *Myo15a*^*sh2/sh2*^; *Overall*< *4 μm n* > *450*: 98 converted of n = 530 for *Myo15a*^*sh2*^/+, 99 converted of n = 450 for *Myo15a*^*sh2/sh2*^). (**j**) Stereocilia isolated from *Myo7a*^*8J*^/+ mouse utricles. (**k**) Stereocilia isolated from *Myo7a*^*8J/8J*^ mouse utricles. (**l**–**n**) Quantitation of utricle stereocilia from 6 *Myo7a*^*8J*^/+ animals and 6 *Myo7a*^*8J/8J*^ animals. (**l**) Number of stereocilia binned by length of stereocilium (1 μm bins); single Gaussian fits. *Myo7a*^*8J*^/+ data were fit with a single Gaussian, R^2^ = 0.9274; data for *Myo7a*^*8J/8J*^ were fit with a sum of two Gaussians. (**m**) Tip signal dependence on length for individual stereocilia. Y-axis is logarithmic, negative values were converted to 0.1 (n = 22 converted of n = 400 total *Myo7a*^*8J*^/+, n = 36 converted of n = 400 total *Myo7a*^*8J/8J*^). Data were fit with linear regression: *Myo7a*^*8J*^/+: 41x − 62 (R^2^ = 0.3549); *Myo7a*^*8J/8 J*^: 34x − 26 (R^2^ = 0.2981). (**n**) Tip signal divided by length (mean ± SEM). Statistical significance determined as in (**h**); ns, not significant. Negative values were converted to zeros (*Overall*: 22 converted of n = 400 for *Myo7a*^*8J*^/+, 36 converted of n = 400 for *Myo7a*^*8J/8J*^; *Overall *> *1* μm: 20 converted of n = 386 for *Myo7a*^*8J*^/+, 33 converted of n = 387 for *Myo7a*^*8J/8J*^). Panel full heights: (**e**, **f**) and (**j**, **k**) 10.1 μm.

In the intact cochlea preparation, the Cy3-ATP tip signal was still visible in *Myo15a*^*sh2/sh2*^ hair cells, but was substantially reduced compared to hair cells of heterozygote siblings (Fig. 5a,b). We also examined cochleas from mice with the 8J allele of *Myo7a*, which have no functional MYO7A^30^. While stereocilia are disorganized in *Myo7a*^*8J/8J*^ mice, the Cy3-ATP tip signal did not appear to be reduced as compared to controls (Fig. 5c,d).

To quantify Cy3-ATP labeling in the mutant mouse lines, we compared stereocilia isolated from utricles of control and mutant mice. Comparing *Myo15a*^*sh2*^/+heterozygote (Fig. 5e) and *Myo15a*^*sh2/sh2*^ knockout mice (Fig. 5f), we measured the length of each stereocilium and the fluorescence intensity at its tip. We plotted the stereocilia length distributions for *Myo15a*^*sh2*^/+and *Myo15a*^*sh2/sh2*^, fitting each with a single Gaussian (Fig. 5g). As expected, *Myo15a*^*sh2*^/+stereocilia were longer (up to 17 μm), while *Myo15a*^*sh2/sh2*^ stereocilia did not exceed 4 μm in length (Fig. 5g). We plotted tip signal against length for each stereocilium, and found that longer stereocilia have higher signal, albeit with considerable variability (Fig. 5h).

To avoid a confound from length, we divided signal by length for each stereocilium, and plotted the mean for heterozygotes and *Myo15a*^*sh2/sh2*^ (Fig. 5i). Even correcting for length, tip signal in *Myo15a*^*sh2/sh2*^ stereocilia was significantly less than in heterozygote siblings. When we only used the heterozygote stereocilia under 4 μm in length to match the length of *Myo15a*^*sh2/sh2*^ stereocilia, a significant difference remained. Since there were fewer heterozygous stereocilia shorter than 4 μm, we added in additional data not plotted in Fig. 5h and increased the number of < 4 μm heterozygote stereocilia to more closely match the number of *Myo15a*^*sh2/sh2*^ stereocilia. In all cases, there was a statistically significant difference between tip signal/length of *Myo15a*^*sh2*^/+ and *Myo15a*^*sh2/sh2*^ (Fig. 5i).

We also isolated stereocilia from *Myo7a*^*8J*^/+heterozygote and *Myo7a*^*8J/8J*^ homozygote mice (Fig. 5j,k) and did not observe any statistical differences between their ratios of tip signal to length (Fig. 5m,n). Interestingly, stereocilia from *Myo7a*^*8J/8J*^ knockout mice were significantly longer than heterozygotes, reaching lengths up to 26 μm (Fig. 5l). The binned distribution appeared to be bimodal, and was better fit with a sum of two Gaussians (Fig. 5l, orange line).

**Figure 6:**
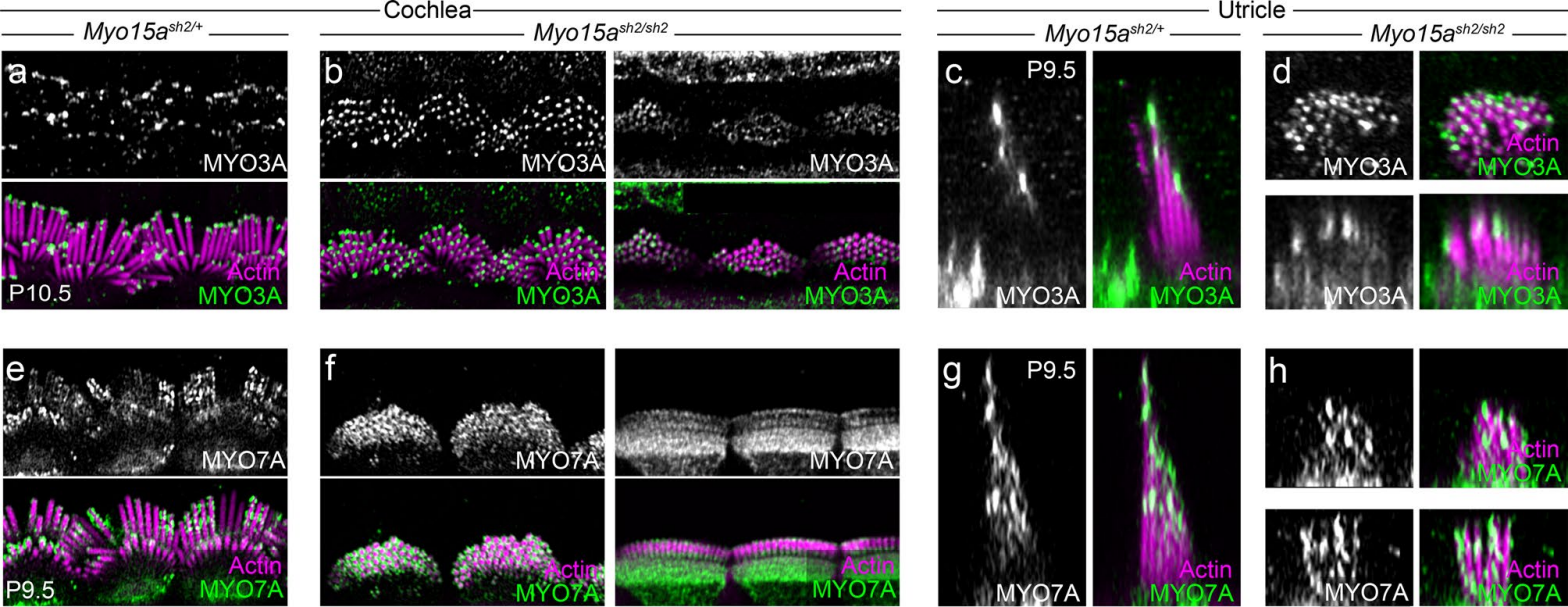
Both MYO3A and MYO7A are normally localized in *Myo15a*^*sh2/sh2*^ mice. All images are Airyscan and are single sections unless stated. (**a**–**d**) IHCs from *Myo15a*^*sh2*^/+ (**a**) and *Myo15a*^*sh2/sh2*^ (**b**) cochleas labeled with anti-MYO3A. Similar results were seen in 1 *Myo15a*^*sh2*^/+ animals and 3 *Myo15a*^*sh2/sh2*^ animals (applies to c-d too). (**c**, **d**) Vestibular hair cells from *Myo15a*^*sh2*^/+ (**c**) and *Myo15a*^*sh2/sh2*^ (**d**) utricles labeled with anti-MYO3A. Viewed in profile using x–z reslice, except for top panels in (**d**). (**e**, **f**) IHCs from *Myo15a*^*sh2*^/+ (**e**) and *Myo15a*^*sh2/sh2*^ (**f**) cochleas labeled with anti-MYO7A. MYO3A. Similar results were seen in 4 *Myo15a*^*sh2*^/+ animals and 4 *Myo15a*^*sh2/sh2*^ animals (applies to g-h too). (**g**, **h**) Vestibular hair cells from *Myo15a*^*sh2*^/+ (**g**) and *Myo15a*^*sh2/sh2*^ (**h**) utricles labeled with anti-MYO7A. Viewed in profile using x–z reslice. Panel full heights: (**a**, **b**) and (**e**, **f**) 23 μm; (**c**, **d**) and (**g**, **h**) 7.8 μm.

MYO3A and MYO3B localize to the tips of stereocilia in hair cells and could plausibly account for the signal remaining in *Myo15a*^*sh2/sh2*^ stereocilia. Because we do not have access to a *Myo3* double knockout line, we instead used an antibody against MYO3A in *Myo15a*^*sh2/sh2*^ mice. In both cochlea (Fig. 6a,b) and utricles (Fig. 6c,d), we observed MYO3A labeling at the tips of stereocilia in heterozygote and *Myo15a*^*sh2/sh2*^ mice. This confirmed that MYO3A is still present in the absence of MYO15A, and that myosin 3 proteins could account for the remaining Cy3-ATP tip signal observed in *Myo15a*^*sh2/sh2*^ mice. As a positive control, we also used an antibody against MYO7A in *Myo15a*^*sh2/sh2*^ mice. We observed that MYO7A label is present even in the absence of MYO15A (Fig. 6e–h).

## Discussion

We present here two methods for visualizing ATP-binding proteins in unfixed hair cell stereocilia, with intact cochlea or isolated utricle stereocilia. Strikingly, the most prominent labeling of stereocilia is of their tips, which is where length is controlled and mechanotransduction occurs. Based on results with mutant mice, we suggest that both methods primarily detect MYO15A.

### Cy3-ATP labels a nucleotide binding site

A large molecule like Cy3-ATP, which contains hydrophilic adenosine triphosphate and hydrophobic Cy3 moieties, conceivably could bind targets in the tissue through the nucleotide or another portion of the molecule. Because unlabeled ADP or ATP completely block labeling, however, it seems very likely that it labels stereocilia through the nucleoside triphosphate moiety. Moreover, the rapid reversibility of labeling is consistent with an interaction through the nucleotide rather than the hydrophobic Cy3 moiety. Finally, the effect of vanadate on the labeling is also consistent with the molecule binding with a bona fide nucleotide binding site.

### Cy3-ATP does not label actin

A plausible scenario for labeling of actin would be for Cy3-ATP to bind to G-actin, presumably during profilin-catalyzed ADP:ATP exchange^31^, followed by addition of the Cy3-labeled monomer to the filament ends growing at stereocilia tips^32,33^. This outcome seems unlikely since two inhibitors of actin polymerization had no effect on the Cy3-ATP tip labeling. An alternative possibility is that Cy3-ATP labels nucleotide-binding sites on newly polymerized actin in filaments, but the persistence of tip labeling after several cycles of washing calls into question why the tips would be preferentially labeled. The evidence makes actin less likely than other molecules, but the high level of actin at stereocilia tips means that it is difficult to rule this possibility out.

### Cy3-ATP labeling of MYO15A

Stabilization of the Cy3-ATP signal at stereocilia tips when vanadate was included in the labeling solution implicates myosin isoforms. Moreover, localization at stereocilia tips is a logical location since most myosins travel towards actin filament plus ends^34^; all stereocilia actin filaments are organized with their plus ends pointing towards stereocilia tips^35^.

The myosin isoforms most convincingly preferentially localized to stereocilia tips are the Class III myosins MYO3A and MYO3B^36–38^, as well as the MyTH4-FERM myosin MYO15A^28,39^. We saw robust labeling for MYO3A in *Myo15a*^*sh2/sh2*^ mice, which nevertheless showed a significant diminution of Cy3-ATP labeling. MYO3A is thus unlikely to account for the majority of the Cy3-ATP tip labeling, MYO7A is often observed at tips, but usually is found throughout the stereocilia^40^ or is concentrated at the upper insertion point of the tip link^7,13^. In addition, Cy3-ATP labeling at stereocilia tips was not diminished in *Myo7a*^*8J/8J*^ mice, which do not express MYO7A^30^. While MYO7A may well be labeled by Cy3-ATP, if so, it is not labeled efficiently.

By contrast, Cy3-ATP labeling decreased significantly (by 50%) in *Myo15a*^*sh2/sh2*^ mice, even when the shorter stereocilia length of the mutants was accounted for. Other myosin isoforms are also not labeled efficiently. MYO6 is by far the most abundant myosin in stereocilia^20^, but is not found at stereocilia tips^19,41^. Interestingly, we often saw lower intensity labeling of Cy3-ATP at stereocilia tapers, where MYO6 is concentrated^19,41^. Like MYO7A, however, if MYO6 is labeled by Cy3-ATP, labeling is not particularly efficient.

In cochlea, the preponderance of Cy3-ATP labeling at stereocilia tips is not stabilized by vanadate. This result suggests the possibility that some of this labeling is not due to myosin molecules. Alternatively, vanadate trapping with Cy3-ATP may be significantly less efficient than trapping with less-bulky nucleotides.

### Comparison to labeling stereocilia myosins using radiolabeled nucleotides

We previously used vanadate and [^32^P]UTP and [^32^P]ATP to label myosin molecules in an isolated stereocilia preparation^9,16^. Under these conditions, a 120-kD protein that we identified as MYO1C was predominantly labeled, although 160 kD and 250 kD proteins were also labeled. The 160 kD protein is most likely MYO6, which is very abundant in vestibular hair bundles^21^, although MYO3A and MYO3B may contribute as well. The 250 kD band labeled by [^32^P]UTP probably corresponds to MYO7A, MYO15A, or both.

While our results suggest that MYO15A is the predominant myosin labeled with Cy3-ATP, they also indicate that other nucleotide-binding proteins are labeled in stereocilia, which may include MYO1C, MYO1H, MYO3A, MYO3B, MYO6, and MYO7A. MYO15A is clearly preferentially labeled, out of proportion with its abundance in stereocilia^21^. Presumably structural characteristics of the active site of MYO15A accommodate the bulky Cy3 moiety in a way that does not occur readily with other myosin isoforms.

### Utility of Cy3-ATP labeling

Cy3-ATP has been used for labeling nucleotide-binding proteins in including muscle or muscle fibers^42,43^, especially for fluorescence resonance transfer experiments, but has not been used for protein localization. Proteins are usually visualized in hair cells using antibodies following fixation, which crosslinks proteins and usually eliminates enzymatic activity. In our permeabilized cell prep, tissue is unfixed and remaining proteins are amenable to chemical intervention. Future modifications of this method should allow us to study myosin ATPase activity in a more-native environment.

Cy3-labeled nucleotides have been used to examine activity of single myosin molecules in vitro^44–47^. We do not have single-molecule resolution in our experiments, however, and it is probable that many myosin molecules contribute to the signal that we measure at stereocilia tips. Nevertheless, we conceivably can use this tip-labeling method to determine the influence of factors that control MYO15A activity or that of other myosins.

## Methods

### Use of live vertebrates

The procedures described in this manuscript were approved by the Oregon Health & Science University Institutional Animal Care and Use Committee (protocol #TR01_IP00000714). All experiments were performed in accordance with relevant guidelines and regulations. Because we used excised explants for investigation, all experiments began with animal euthanasia, which followed guidelines reported in the American Veterinary Medical Association (AVMA) Guidelines for the Euthanasia of Animals (2020).

We used C57BL/6J mice (RRID:IMSR_JAX:000664, Jackson Laboratories, Bar Harbor, ME), *Myo15a*^*sh2/sh2*^ on a C57BL/6 background (MGI Cat# 3046320, RRID:MGI:3046320), and *Myo7a*^*8J/8J*^ on a C57BL/6 background (MGI Cat# 5425619, RRID:MGI:5425619)^30^. Mice were backcrossed beyond the 6th generation. In all cases, we used a mixture of female and male animals.

When possible, we complied with ARRIVE guidelines^48^. In the experiments of Figs. 2 and 4, only C57BL/6J mice were used in comparisons of Cy3-ATP labeling conditions; in addition, these experiments were qualitative and not quantitative. Quantitative experiments of Fig. 3 also used C57BL/6J mice. In the experiments of Figs. 5 and 6, we compared either *Myo15a*^*sh2*^/+heterozygotes to *Myo15a*^*sh2/sh2*^ homozygotes (both Figs. 5 and 6) or *Myo7a*^*8J*^/+heterozygotes to *Myo7a*^*8J/8J*^ homozygotes (Fig. 5 only). Animal numbers are indicated in figure legends; power analyses were not used to determine sample sizes. No results were excluded for quantitative analysis. Randomization was not used. Blinding was impossible in the comparisons of different mouse genotypes as the genotypes were obvious from phenotype. Outcome measures and statistical methods for quantitative experiments (Fig. 5) are discussed below.

## Materials

EDA-ATP-Cy3 (Cy3-ATP) was obtained from Jena Bioscience (#NU-808-CY3); sodium orthovanadate (referred to as vanadate) was purchased from Sigma-Aldrich (#S6508); 6-(2-MeBu)-ADP (referred to as ADP) was purchased from Biolog (#M028). Latrunculin A was obtained from Cayman Chemical Company (#10010630) and cytochalasin D was obtained from ApexBio (#B6645). Alexa Fluor 488 phalloidin (#A12379; phalloidin-488) and Alexa Fluor 568 phalloidin (#A12380; phalloidin-568) were obtained from Thermo Fisher Scientific.

Primary antibodies for immunocytochemistry were 1:500 mouse anti-MYO7A (Proteus, Ramona, CA; #25-6790; RRID:AB_10015251) and 1:250 mouse anti-MYO3A (QHF antibody, raised against the C-terminal 22 amino acids of *X. laevis* MYO3A; from B. Burnside, University of California, Berkeley). The secondary antibody was Alexa 488 donkey anti-rabbit IgG (H + L) from Thermo Fisher (#A-21206).

### Permeabilized cell preparation

Using forceps, we dissected cochleas or utricles in cold Hank’s balanced salt solution (#14025076, Thermo Fisher Scientific) supplemented with 5 mM HEPES, pH 7.4 (dissection buffer). In cochleas, we removed the modiolus and the basilar membrane before permeabilizing for 10 min with gentle rotating in intracellular solution (15 mM HEPES, 50 mM KCl, 1 mM MgCl_2_, 1 mM EGTA, 1 mM DTT, pH 7.5) with 0.008% saponin and 0.012% or 0.016% Triton X-100 (higher concentrations for P6.5 and older). To label actin filaments, we also added 1:100 phalloidin-488 to the permeabilizing solution.

After washing three times with intracellular solution, we mounted the cochleas on a slide with the hair cells facing up and removed the tectorial membrane. Slides were prepared with two strips of 0.12 mm SecureSeal Adhesive (Grace Biolabs SA-S-1L, #092321) so that, after addition of a 50 × 22 mm coverslip, the tissue was inside a handmade perfusion chamber (Fig. 1b).

For utricles, we used an eyelash brush to remove otoconia, and then gently blotted the utricles against coverslips to isolate stereocilia as previously described^19,49^. Square #1.5 glass coverslips (Corning) were washed with water and 70% ethanol, autoclaved, and then coated with 100 μg/ml poly-l-lysine for 10–20 min. Poly-l-lysine was removed and coverslips dried for 30–60 min. The coverslip was wetted with intracellular solution, then utricles were gently pressed against the poly-l-lysine-coated coverslip surface to isolate stereocilia. We then permeabilized the blotted stereocilia for 5 min to intracellular solution with 0.008% saponin, 0.012% Triton X-100, and 1:100 Alexa Fluor 488 phalloidin. After washing three times with intracellular solution, we attached coverslips face-down to slides with two strips of SecureSeal Adhesive, taking care to renew the solution during the preparation steps to prevent blotted stereocilia from drying out.

After mounting the perfusion chambers on the microscope, solution was placed on one side of the overhanging coverslip. When put in contact with the solution in the chamber on the other side of the coverslip, a piece of tissue paper wicked the old solution out and perfused the new solution into the chamber by capillary action (Fig. 1c). Given the chamber size, 50 µl was sufficient to replace the solution, and up to 200 µl could fit on the coverslip overhang.

Samples were labeled with internal solution containing 5 µM Cy3-ATP; in some cases, 1 mM vanadate was included as well. To avoid polymerization of vanadate, 200 mM stock solutions at pH 10 were prepared from sodium orthovanadate^17^ and stored at − 80 °C.

### Microscopy

Figure 1b,c was captured using an iPhone 5 camera. Figure 2a was captured using a Zeiss Elyra 7 inverted microscope using lattice-based SIM with a 63 × 1.4 n.a. Plan Apochromat objective, and processed using automated Wiener filter estimation determined by the manufacturer's algorithm. We omitted the default baseline cut and manually assessed the lower cut-off to preserve faint signals in high dynamic range images. All other images in the paper were captured with an LSM 880 using Airyscan, and processed using automated Wiener filter estimation determined by the manufacturer’s algorithm. Permeabilized isolated stereocilia from utricles and fixed tissues were imaged using a 63 × 1.4 n.a. Plan Apochromat oil objective, while permeabilized cochleas were imaged using a 40 × 1.2 n.a. W LD LCI Plan Apochromat water objective. Figure 2f used a 20× water objective.

### Quantification of Cy3-ATP signal

We used Fiji/ImageJ to quantify fluorescence signal and stereocilia length. We used GraphPad Prism to generate all graphs and perform all statistical tests.

In Fig. 3c, we quantified Cy3-ATP signal at the stereocilia tips of the cell from Fig. 3a,b. To do this, we generated z-projections using averaged intensity for 12 time points, the first being during exposure to Cy3-ATP, and the next 11 time points being post-wash images every minute for 10 min. We used the phalloidin signal as a reference point to maintain the same z-depth of the sections used for the projection. At each time point, for both conditions (with or without vanadate), we drew seven regions of interest (ROIs) around the same seven stereocilia in row 1. These stereocilia were chosen because they were more central and were well isolated from each other and objects in the image. We measured the integrated density in each ROI of the channel with an emission peak at 568 nm. We also used collected integrated density from an area adjacent to the stereocilia tips to subtract background. We then graphed the mean with standard deviation of each time point for the with or without vanadate conditions (Fig. 3c). We compared the two conditions at each time point; statistical significance was determined by multiple t-tests with a two-stage step-up method^50^, with Q = 1%. We used G*power to determine that we had reached appropriate sample size to detect statistical differences with α = 0.05, which was a minimum of three stereocilia in each condition.

In Fig. 5, we analyzed images of stereocilia that had been captured after exposure to 5 μM Cy3-ATP and 1 mM vanadate, and then washed with intracellular solution containing 1 mM vanadate. Because vanadate extended the life of the signal, images were taken up to 1 h post-wash for each utricle blot. To quantify tip signal, we drew an ROI around the tips of stereocilia and measured the integrated density of the channel with an emission peak at 568 nm. We also measured the length of each stereocilium. We subtracted background by measuring integrated density in > 10 ROIs drawn over blank areas of the image, avoiding large fluorophore aggregates. To avoid sample bias, we measured all stereocilia whose lengths could be determined and whose tips were not confounded by fluorophore aggregates or other background noise. Then we chose a random sample of 50 stereocilia from each utricle. Data obtained for statistical analysis of *Myo7a*^*8J*^/+ and *Myo7a*^*8J/8J*^ labeling were each from N = 4 mice, 8 utricles, and n = 400 stereocilia. Data for *Myo15a*^*sh2*^/+ used N = 3 mice, 6 utricles, n = 300 stereocilia; *Myo15a*^*sh2/sh2*^ used N = 5 mice, 9 utricles, n = 450 stereocilia. For Fig. 5g,l, we binned stereocilia by length and plotted the number of stereocilia in each 1 μm bin. We fitted a Gaussian (in the case of *Myo7a*^*8J/8J*^, a sum of two Gaussians) to the data for each genotype. For Fig. 5h,m, we plotted stereocilia length against stereocilia tip signal, then fitted a linear regression to the data points for each genotype. For Fig. 5i,n, we divided stereocilia tip signal by stereocilia length for each individual stereocilium in the dataset, and then graphed the mean and SEM. For Fig. 5i, negative values were first converted to zeros. *All Fig. **5**i data:* 35 converted of n = 298 for *Myo15a*^*sh2*^/+, 99 converted of n = 450 for *Myo15a*^*sh2/sh2*^; *Fig. **5**i data *< *4 μm*: 25 converted of n = 143 total *Myo15a*^*sh2*^/ + , 99 converted of n = 450 for *Myo15a*^*sh2/sh2*^; *Fig. **5**i data *< *4 μm n* > *450*: 98 converted of n = 530 for *Myo15a*^*sh2*^/+, 99 converted of n = 450 for *Myo15a*^*sh2/sh2*^. For Fig. 5n, negative values were converted to zeros. *All Fig. **5**n data*: 22 converted of n = 400 for *Myo7a*^*8J*^/+, 36 converted of n = 400 for *Myo7a*^*8J/8J*^; *Fig. **5**n data *> *1* μm: 20 converted of n = 386 for *Myo7a*^*8J*^/+, 33 converted of n = 387 for *Myo7a*^*8J/8J*^). Statistical significance was determined by multiple t-tests with a two-stage step-up method^50^, with Q = 1%.

### Immunocytochemistry

Using forceps, we dissected cochleas or utricles in dissection solution. For utricles, we used an eyelash brush to remove otoconia prior to fixation. Tissue was fixed for 1 h at room temperature in dissection solution with 4% paraformaldehyde, then washed 2–3 times with PBS. Tissue was then permeabilized for 10 min with gentle rotating in PBS with 0.2% Triton X-100, and then blocked for 2 h at room temperature with blocking buffer. Primary antibody was applied in blocking buffer overnight at 4 °C, on a rotator, washed 3× in PBS, then phalloidin and secondary antibody were applied for 4–5 h at room temperature in blocking buffer. We used Alexa-488 secondary antibodies at 1:1000, and phalloidin-568 at 1:500 to stain actin. After washing 3× in PBS, we mounted the tissue in (mounting solution). The tectorial membrane was removed from cochlea while mounting, and utricle mounting used a spacer.

## Data Availability

Figure source data generated during the current study are available from the corresponding author on reasonable request.

## Abbreviations

Cy3-ATP: γ-(6-Aminohexyl)-ATP-Cy3
IHC: Inner hair cell
OHC: Outer hair cell
